# Giant laterocervical fibrosarcom

**Published:** 2009

**Authors:** C Nistor, M Davidescu, O Rus, B Marinescu, Ioana Ştefănescu, A Tudose, T Horvat

**Affiliations:** * Central Clinical Military and Emergency Hospital Thoracic Surgery Clinic; ** Central Military Clinical and Emergency Hospital Plastic Surgery and Reconstructive Microsurgery Compartment; *** Central Clinical Military and Emergency Hospital Pneumology Department; **** Central Clinical Military and Emergency Hospital Anesthesia and Intensive Care Department

**Keywords:** laterocervical tumor, fibrosarcoma, skin graft

## Abstract

Soft tissue sarcomas are rare tumors representing 1% of all malignancies and less than 10 % concerning head and neck tumors. We are presenting the case of a 42 year old patient that was admitted in our service for a giant laterocervical tumor (15/12/10 cm). We performed total excision of this tumor en bloc with the involved tegument; the resulting defect was covered with a split thick skin graft. The weight of the tumor was 500 g. Histopathological examination revealed an intermediate-grade fibrosarcoma. The postoperative evolution was good; radiotherapy was indicated.

## Introduction

Soft tissue sarcomas are rare tumors representing 1% of all malignancies and less than 10 % concerning head and neck tumors. [**1**]. Cervical localization raises surgical technical problems because of the proximity of a lot of important structures.

## Case presentation

**Fig. 1:**
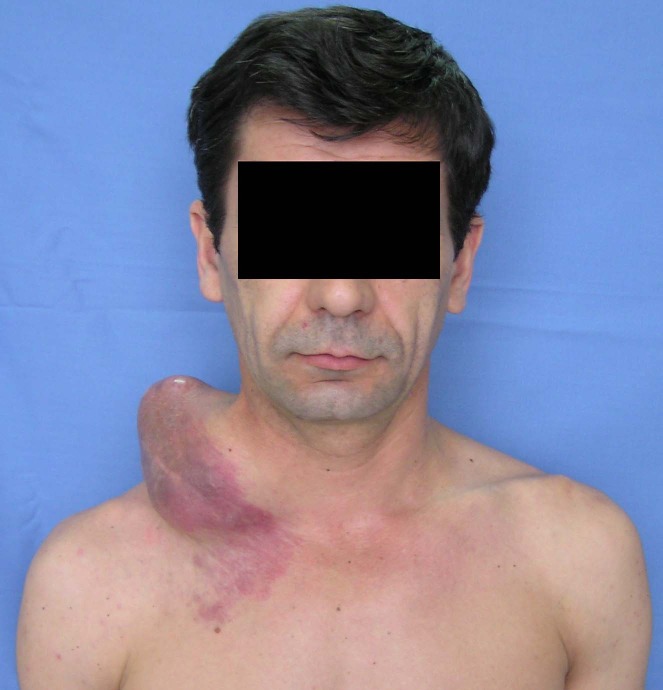
Giant right laterocervical tumor

We are presenting the case of 42 year old man admitted in the Thoracic Surgery Department of the Central Military Emergency Hospital with a tough, painless, right laterocervical tumor (**Fig. 1**), measuring 15/12/10 cm, which appeared last year with progressive growth especially in the late two months.

In 2004 he had a previous surgical procedure for a laterocervical tumor considered a lipoma, in the absence of histopathologic examination. 

Clinical examination revealed a giant, firm, laterocervical tumor, relatively mobile on profound plans but infiltrating the tegument.

At high resolution computed tomography the laterocervical tumor was heterogeneous, oval-roundish, with a density of 30 UH and 10.4 / 7.3 / 7.2 cm in size. 

**Fig. 2:**
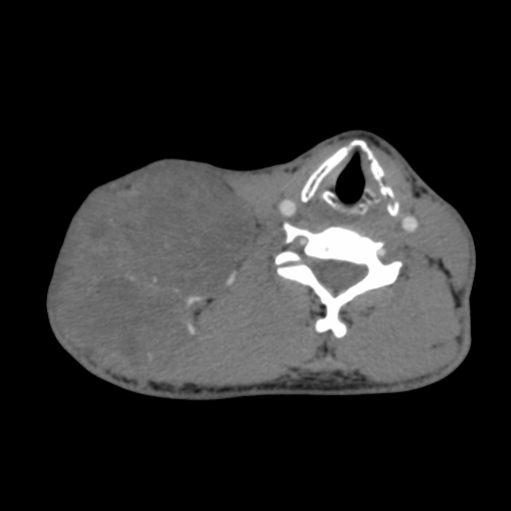
CT – laterocervical tumor

**Fig. 3:**
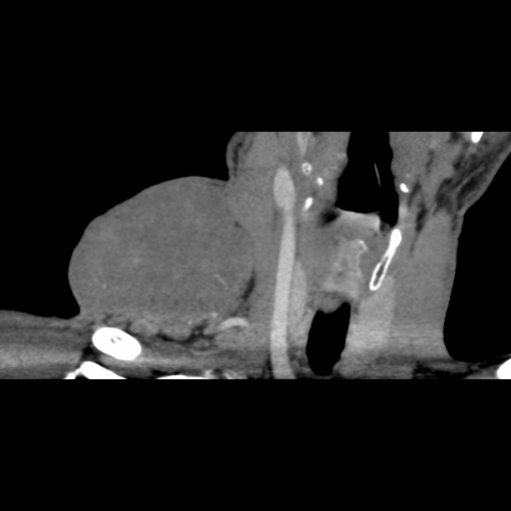
Transversal CT reconstruction

After intravenous administration of contrast substance, the tumor had a moderate iodophilia, with central necrosis areas, other areas with low density, and no contact with large vessels. There was a wide vascular network that penetrated the tumor; some vessels were branches of the thyreocervical trunk, which was in contact with the inferior tumor border. In front of the tumor there was the sternocleidomastoidian muscle, the anterior and middle scalene muscles. Posteriorly there were the trapeze and elevator scapulae muscles with no limit between them and the tumor. The lower tumor border was near clavicle and the anterior and lateral deltoid muscles. The subcutaneous fat tissue was infiltrated (**Fig. 2**, **Fig. 3**).

The sanguine circulation in right commune, internal carotid and vertebral artery was not modified at Doppler ultrasound examination. The jugular vein was compressed. The lower border of the tumor was near the subclavian artery. The thyreocervical trunk had lost its normal shape because of the tumor compression and the inclusion of the lower thyroid artery.

The tumor giant size and the tegumentary involvement made necessary a multidisciplinary approach by a team of thoracic and plastic surgeons.

**Fig. 4:**
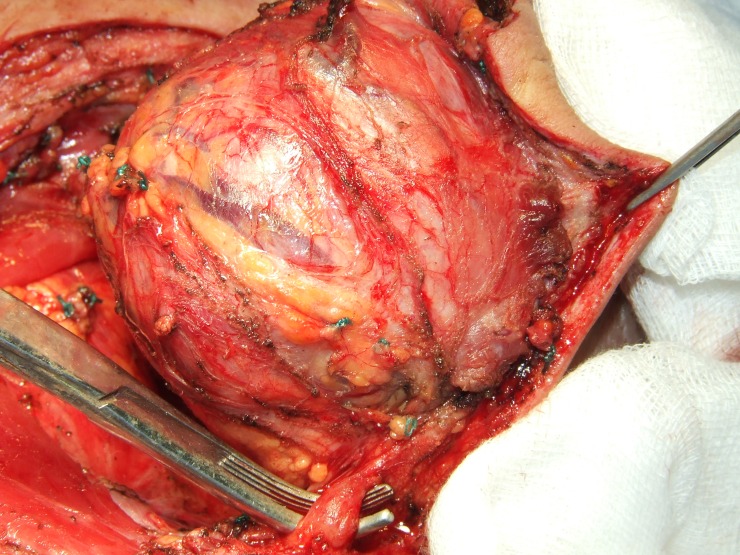
Surgical aspect

**Fig. 5:**
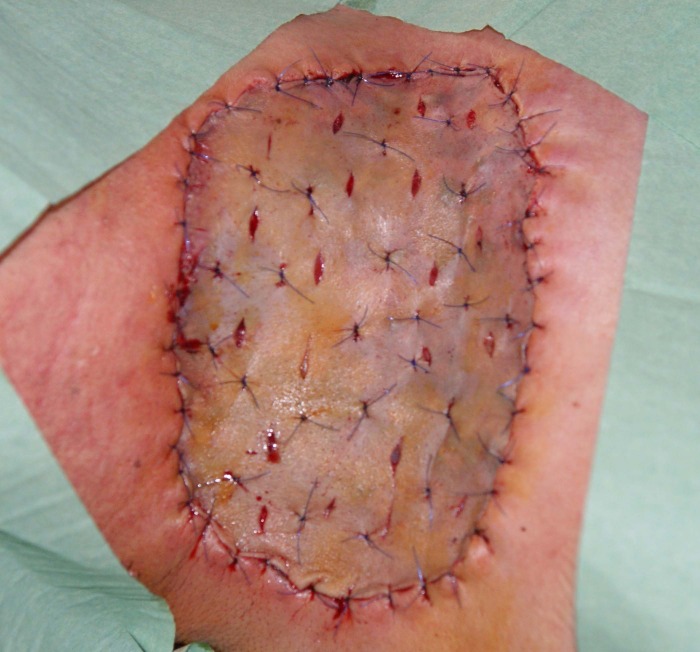
Immediately after surgery

**Fig. 6:**
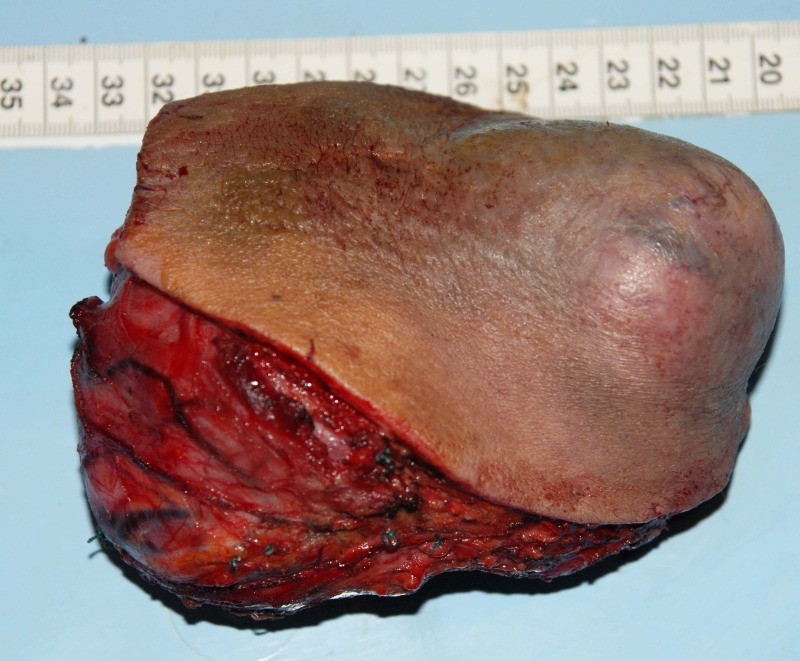
Surgical specimen

We made a fusiform incision into the healthy tegument passing through the lateral neck triangle delimitated by the trapeze muscle and the posterior limit of sternocleidomastoidian muscle. The tumor appeared to be formed by multiples lobes and was covered by a pseudo capsule with large blood vessels coming from different sources (**Fig. 4**). The vessels had high caliber but the large neck vessels weren’t invaded. The muscles involved with the tumoral tissue were: platisima, omohioidian, posterior limit of sternocleidomastoidian and scapula elevator muscle. The tumorectomy was made en bloc with infiltrated skin until healthy tissue was reached. The infiltrated external jugular vena was anatomized and ligated. The resulting tegumentary defect was covered with split thick skin graft harvested from the right thigh (**Fig. 5**). The skin graft was sutured on the recipient zone with separate surgical 3.0 lines. The surgical specimen weighed 500 g (**Fig. 6**).

**Fig. 7:**
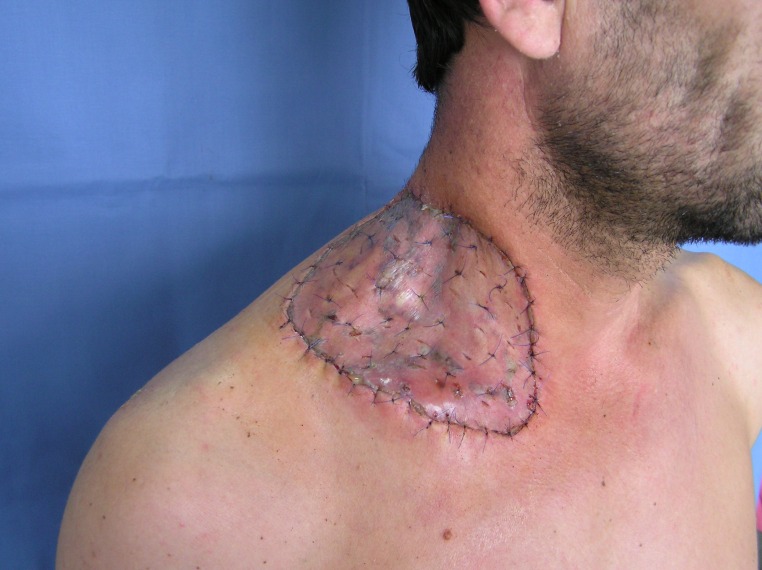
Seven days after surgery

**Fig. 8:**
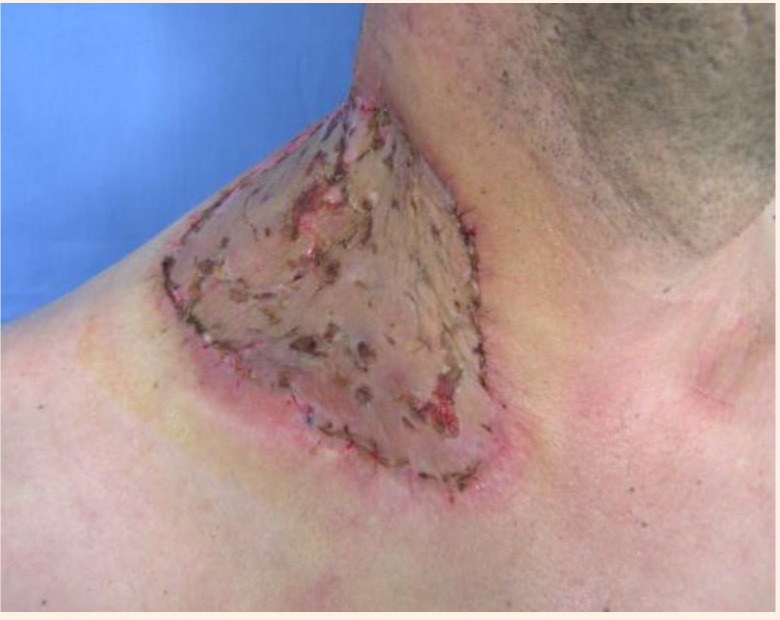
Fourteen days after surgery

The local evolution was very good with an integrated skin graft rate of 98% after 14 days (**Fig. 7**, **Fig. 8**).

Histopathological examination described: a tumor partially covered with infiltrated tegument and a fibrous capsule penetrated by tumor tissue, comprised of fusiform, oblong and thin cells, arranged in an interlocking pattern, with frequent mitosis - histological findings compatible with an intermediate grade fibrosarcoma.

Fibrosarcoma is a malignant tumor of mesenchymal origin composed of malignant fibroblasts and collagen fibers. It is most frequently encountered between 35 and 55 years of age [**2**] and has an equal repartition on both sexes [**3**]. 

The specific causes for the apparition of this tumor are not known, they are associated with genetic mutations, inherited syndromes like neurofibromatosis, scars, carcinogenic substances, irradiation [**2**][**3**].

Fibrosarcomas most commonly manifest as painless, gradually enlarging masses.

Radiological examination of the affected zone reveals the sizes, eventual calcifications, bone involvement and the pulmonary radiography, the presence of secondary lesions.

High resolution computed tomography identifies the specific tumor sizes and local extension, the presence of secondary lesions.

The best imaging investigation for soft tissue tumors is MRI. It provides information about the size of the lesion, tumor structure, local extent, neural and vascular involvement.

Ultrasonography gives information about the localization, size and structure of the tumor and using Doppler investigation, about its vascularization and large vessels compression. 

Scintigraphy can reveal hypercaptation in soft tissues sarcomas and secondary bone involvement.

Selective angiography shows the vessels network, if an important branch is affected and permits artery embolisation or local chemotherapy administration [**3**].

The main therapy for soft tissue sarcomas consists of complete surgical excision with a cuff of normal tissue. In the case of tumors of the head and neck this is difficult to achieve because of the proximity of large vessels and other important structures [**5**]. Radiotherapy is used for local tumor control and can reduce the frequency of metastatic disease. The use of chemotherapy is controversial, but it can be considered in case of secondary lesions.

Local tumor recurrence is observed in 60% of cases. This raises the importance of local radiotherapy, it is demonstrated that it reduces the chance of tumor relapse with 25% [**2**]. In case of local recurrence there is a higher probability of secondary lesions development. After 5 years, 60% of the patients present metastases [**3**]. The lung is the most frequent target for secondary determinations [**5**]. In the absence of pulmonary involvement the existence of other metastasis is highly unlikely to appear [**1**].

The prognosis is determined by: tumor size and extension, histological grade, the possibility of large resection in healthy tissue. The survival rate at 5 years is 40-60%.

Skin grafting was used for the first time by Reverdin who performed the first skin transplant in 1870. Skin grafts are used to provide coverage over a broad spectrum of open soft tissue defects. Those are transferred from a donor region to a receptor one of the same subject or to a different person.

According to their thickness, skin grafts are divided in split thickness and full thickness. The former is further divided into thin, intermediate and thick.

The presented case is special due to the giant size of the tumor, for this region, the long period of time between the emergence of the tumor and hospital presentation which emphasizes the necessity and the importance of medical education. The National Institute for Health and Clinical Excellence (NICE) from Great Britain 2005 guide to general practitioners emphasises that: *“In patients with an unexplained lump in the neck which has recently appeared or a lump which has not been diagnosed before that has changed over a period of 3 to 6 weeks, an urgent referral should be made”* [**4**].

In this case we used an intermediary split thick skin graft with a thickness of 0.38 mm harvested with an electrodermatome from the anterior side of the right thigh. This skin graft contains: epidermis, papillary layer and varying portions of the underlying dermis. We chose this type of skin graft because it is well integrated on clean receiver surfaces permitting a rapid and well recovery. If the tumor recidivates, the intermediary skin graft thickness allows a quick tumoral tissue observation and gives the surgeon the opportunity to perform further excision.

After the patient’s complete local healing radiotherapy is recommended. He will be monitored monthly and then, every 3 or 6 months for a minimum of 5 years.
